# F169. BRAIN CONNECTIVITY DURING PSYCHOLOGICAL STRESS IN PATIENTS WITH SCHIZOPHRENIA

**DOI:** 10.1093/schbul/sby017.700

**Published:** 2018-04-01

**Authors:** Mariana Castro, Gabriela De Pino, Hernán Bocaccio, Stella Sanchez, Lucas Drucaroff, Elsa Costanzo, Agustina Wainsztein, Salvador M Guinjoan, Mirta F Villarreal

**Affiliations:** CONICET - UBA - FLENI

## Abstract

**Background:**

It is commonly accepted that in most patients with schizophrenia external factors act on genetic predisposition to produce active psychotic symptoms. In fact, we showed that patients with schizophrenia have an abnormal brain activation and peripheral autonomic response to psychological stress. We sought to characterize the brain connectivity networks of such response in schizophrenia.

**Methods:**

We studied the pattern of brain connectivity in relation to mental arithmetic stress paradigm in 21 patients and 21 healthy subjects aged 18 to 50 years, using 3T-fMRI. A period of 6 minutes of resting state acquisition (PRE) were followed by a block design with three 1-minute CONTROL task (one digit sum), 1 minute STRESS task (two digit subtraction) and 1 minute rest after task (POST). Pairwise Pearson correlations were calculated between 90 regions of interest. Data were analyzed with MATLAB and SPSS software.

**Results:**

Patients with schizophrenia showed a lower connectivity network between fronto-temporal limbic areas compared with control subjects during control and stress task.

Moreover, we observed a great variability of link density during resting state in patients but not in controls, and it diminishes in response to task.

**Discussion:**

Patients present abnormalities in networks related to stress response showing an alteration in fronto-temporal connectivity, and a poor and random modulation of these networks at rest. Current and previous findings suggest abnormal fronto-temporal connectivity that ultimately would lead to psychotic symptoms emergency in response to an environmental stressor and, even, could be related to hypervigilance and misattribution feeding into the paranoid cognition characteristic of patients with schizophrenia.

